# Infection after surgical implant generation network (SIGN) nailing in treatment of long bone shaft fractures in Ethiopia: analysis of a 4-year results

**DOI:** 10.1007/s00590-022-03454-1

**Published:** 2022-12-07

**Authors:** Birhanu Beza, Almaw Bitew, Debas Yaregal Melesse

**Affiliations:** 1 Department of Orthopaedic Surgery, College of Medicine and Health Sciences, Bahir Dar University, Bahir Dar, Ethiopia; 2 Department of Orthopaedic Surgery, College of Medicine and Health Sciences, University of Gondar, P. O. Box 196, Gondar, Ethiopia; 3 Department of Anaesthesia, College of Medicine and Health Sciences, University of Gondar, P. O. Box 196, Gondar, Ethiopia

**Keywords:** Factors, Infection, Long bone fracture, Prevalence, SIGN nail

## Abstract

**Background:**

One of the challenge to manage long bone fracture is the risk of infection. Intramedullary nailing is the standard treatment of long bone shaft fractures. Infection from the surgical site during orthopedic management is posing postoperative burdens in different perspectives like patient perspectives and healthcare facilities. However, there is limited information on the magnitude of infection in Ethiopia after surgical implant generation network (SIGN) nailing in the treatment of long bone shaft fractures. Therefore, the current study aimed to assess the prevalence of infection in patients with long bone shaft fractures treated with surgical implant generation network (SIGN) nailing.

**Objective:**

To assess prevalence of infection in patients with long bone shaft fractures treated with SIGN nailing at Felege Hiwot Referral Hospital from January 1, 2015, to December 31, 2018, Bahir Dar, Northwest, Ethiopia.

**Methods:**

This was a retrospective study over a period of 4 years. SIGN surgical-related data, presence or absence of infection from the documented information were collected from the chart/the source. The types of infection were also collected with the standard classification as superficial, deep and deep with osteomyelitis. Age, sex, fracture pattern, nature of fracture, mechanism of injury, prophylaxis antibiotics, nail type, follow-up in weeks and other factors were also extracted from the patients’ charts with structured checklist. Data were analyzed with statistical package for social sciences (SPSS) version 23. The analyzed data were presented with texts, tables and a graph.

**Results:**

Three hundred and eighty-two long bone fractures were treated by locked SIGN intramedullary nailing during the study period. After screening the inclusion criteria, a total of 311 cases were included in this study. A total of 13 (4.2%) patients who treated with SIGN intramedullary nailing developed infection.

**Conclusions:**

We conclude that the overall prevalence of infection with SIGN intramedullary nailing is almost similar with the surgeries done in the developed countries.

## Introduction

Long bone fractures can be resulted from high-energy trauma, like road traffic accidents, falls from a height, and gunshot injuries which may result into comminuted or segmental fractures [1]. Open fractures are unique in the urgency they impart to the injury and the therapeutic challenge they pose [2]. Fractures following high‑energy trauma are often associated with extensive injuries [3]. Healing from fractures is determined by the situation of the surrounding soft tissues and the local blood supply to the bone [4]. A substantial amount of energy is transferred to the limb leading to damage of both the soft tissue envelope and the bone [5]. Road traffic accident is the leading cause of injury, resulting in large number long bone fractures in Ethiopia [6].

Management of open long bone fracture is always challenging for orthopedic trauma surgeons, due to different associated factors like osteosynthesis-associated infection (OAI) [7]. The surgeon faces difficulty to manage when complex fracture is associated with a significant open soft tissue damage of skin, muscles and neurovascular structures [8]. Periosteal stripping and damage to the soft tissue envelope are key factors for most common complications of open fractures like infection [9]. Urgent and thorough soft tissue debridement, proper surgical fracture stabilization as well as the administration of intravenous and local antibiotics as adjunctive therapy are mandatory to reduce the risk of infection [10]. A study conducted on intramedullary nailing of open and closed tibial shaft fractures among 551 cases showed that as part of the post procedural complications, infection was the leading complication (6.7%) [11].

There are a number of options for the treatment of long bone fracture starting from non-touch conservative treatment, external fixator to internal fixation with locked intramedullary nails [12, 13]. There is a consensus to treat Gustilo-Anderson grades I, II, IIIA and all closed long bone fractures with locked nails, while external fixation is reserved for IIIB and IIIC open fractures [14]. Damage control orthopedics (DCO) can be practiced by putting initial external fixator and then apply intramedullary locking nail ones the patients clinical condition is stable [13].

Intramedullary (IM) nailing is considered the gold standard of treatment of closed and many open femoral, tibial and humeral shaft fractures due to its biomechanical and biological advantages [15]. IM nail was introduced by Gerhard Küntscher in 1939 and remains the method of choice for treating long bone fractures [5]. Major complications following this procedure include infection, compartment syndrome, venous thromboembolic events, fat embolism syndrome, neurovascular damage and nonunion [16]. In a comparative study, the incidence of deep and superficial infections, local implant irritations and secondary procedures in the percutaneous locked plate group was greater than those in the intramedullary nail group [17]. Surgical implant generation network **(**SIGN) nail was introduced by Zirkle in 1999 [12]. This nail is gaining universal acceptance in developing countries due to its ease of use without the need for an image intensifier [18]. The surgical implant generation network (SIGN) supplies intramedullary (IM) nails for the treatment of long bone fractures free of charge to hospitals in low- and middle-income countries (LMICs) [19]. Since January 2008, it has become possible to achieve interlocking nail insertion in Ethiopia, because of SIGN (surgical implant generation network), Richland, WA, USA, who has provided to the country physician with training and equipment, interlocking intramedullary nails with interlocking screws [6]. Most operations are reported to the SIGN Online Surgical Database (SOSD) [20]. Based on the information obtained from the SOSD, there are low infection rates and suggesting that IM nailing is a safe procedure in low- and middle-income countries [21].

The operative fixation of skeletal fractures can be highly complex due to the unpredictable nature of the bone damage, and the magnitude of concomitant injuries that may need to be considered and the frequency of life-threatening situations in emergency care [22]. The term osteosynthesis-associated infection (OAI) is defined as the clinical or subclinical infection of a fracture following surgical fixation with an internally placed implant [23]. The IM canal is surgically less accessible, and diagnosis is more likely to be delayed [24].

Surgical site infection in orthopedic implants is a major problem, causing long hospital stay, cost to the patient and is a burden on healthcare facilities [25]. Early infection after open reduction and internal fixation (ORIF) of a limb bone is defined as bacteriologically documented, deep and/or superficial surgical site infection (SSI) diagnosed within 6 months after the surgical procedure [26]. It increases rate of nonunion, osteomyelitis, implant failure, sepsis, multiorgan dysfunction and even death [27]. Antibiotics cannot cross this film to reach the bacteria’s, causing infection [28]. The prevalence of infection following internal fixation of fractures is approximately 5% overall but may exceed 30% in open fractures [29]. The risk of infection following IM nailing of closed long bone fractures is thought to be similar to the general risk of infection after any orthopedic trauma procedure, but this risk is substantially increased in the setting of open fractures and has been reported to range between 4 and 7% [16].

Fractures of long bones can be treated conservatively with splinting, casting, traction or with external fixation, but these methods are associated with several complications such as malunion, pin tract infection, nonunion and joint stiffness [30]. A strong argument against the use of modern orthopedic surgical trauma care, apart from the cost of the implants and the lack of skilled personnel, has been the fear of infection with regarded some surgeons have believed that internal fixation of fractures carries too high a risk of infection in low-income countries (LICs) to merit its use there [20].

However, there is limited information on the magnitude of infection in Ethiopia after surgical implant generation network (SIGN) nailing in the treatment of long bone shaft fractures.

Therefore, the current study aimed to assess the prevalence of infection in patients with long bone shaft fractures treated with surgical implant generation network (SIGN) nailing.

## Objectives of the study

### General objective

o To determine prevalence of infection and associated factors in patients with long bone fractures treated with SIGN nail at Felege Hiwot Referral Hospital (FHRH), Bahir Dar, Northwest Ethiopia.

### Specific objectives

o To assess prevalence of infection in patients with long bone fractures treated with SIGN nail.
o To identify associated factors of infection in patients with long bone fractures treated with SIGN nail.

## Methods

### Study area and period

The study was conducted from January 1, 2015 to December 31, 2018 at Felege Hiwot Referral Hospital (FHRH) in Bahir Dar City, Amhara Regional State, Northwest, Ethiopia. Bahir Dar is the capital city of Amhara National Regional State, located 565 km Northwest of Addis Ababa with an altitude of 1799 m above sea level with warm and temperate climate with population of 297,775 of which 156,515 are females as per 2015 census. FHRH is one of the 42 governmental hospitals in Amhara Regional State with estimated catchment population of seven million and has eight cluster primary hospitals and four health centers. The hospital serves for more than 5,000,000 populations in its catchment area. The hospital has one big orthopedics trauma ward which possesses around 54 beds and three major operation theatres.

#### Study design

A hospital-based retrospective cross-sectional study design was employed.

#### Source population

All patients with long bone fractures who had locked SIGN intramedullary nail from January 1, 2015, to December 31, 2018, at Felege Hiwot Referral Hospital Bahir Dar, Northwest Ethiopia.

#### Inclusion criteria

Patients with long bone fractures admitted to the orthopedic surgery department or presented with nonunion and operated with SIGN implant, patients whose operations notes were fully documented on the chart or obtained from electronic data from SIGN online surgical database**,** whose follow-up status was clearly known (clearly documented on follow-up database or chart), both standard nails and/or Fin nails were included as SIGN nail cases, and fractures in bilateral and floating knee cases were included in the current study.

#### Exclusion criteria

Incomplete data in the SIGN surgical data source and those who had lost follow-up**,** patients with other systemic disease leading to increase susceptibility to infection like uncontrolled diabetes, HIV and otherimmunocompromisedconditions, patients who were operated with intramedullary nailing other than SIGN nail, patients with SIGN nails done in other hospitals but were in follow-up in FHRH, SIGN nails used other than long bone fracture fixation operations; for instance, joint fusion, and SIGN nails used for the indication of deformity or shortening in a completely healed fracture.

### Sample size calculation

A survey of patients with long bone shaft fractures who had SIGN nail with the availability of necessary information in line with inclusion and exclusion criteria was selected and included in the final analyses.

### Study variables

#### Independent variables

Age, sex, cause of fracture, severity of injury, nature of fracture, duration of presentation and antibiotics.

#### Dependent variables

Prevalence of infection after long bone fracture treated with SIGN nail.

#### Data collection tool

The medical document and SIGN surgical data source over the data collection period were used to gather the data. Using structured checklist, information on patients’ socio-demography, booking, and clinical features at presentation were extracted. Two orthopedics residents and two general practitioners were the data collectors. Checklist was checked by data collectors and supervisor on daily basis for completeness, clarity and meaning fullness.

### Data processing, analysis and quality control

Data were taken from SIGN surgical data source. After data collection, the data were coded, entered and stored to SPSS version 23 software packages for final analysis. Descriptive statistics such as mean, percentage and standard deviation were computed and presented with texts, tables and a graph.

Prior to data collection, the checklist was tested on 5% of samples to check the consistency of the checklist format and the ability of the data collector’s performance based on the checklist. The checklist was modified based on the pretest results. One day training and orientation on how to carry out data collection and quality control were given for the data collectors.

### Ethical clearance

Ethical approval was asked and was cleared from the ethical committee of college of medicine and health sciences; Bahir Dar University. Permission was obtained from SIGN surgical data program office for Bahir Dar University. Confidentiality was maintained when handling each case files.

## Results

Three hundred and eighty-two (382) patients were operated with SIGN intramedullary nail in the study period, of which 311 patients were included in the study. Seventy-one patients were not included in the final analyses because of having incomplete data. Two hundred and eighty-nine (92.9%) were ordinary SIGN nails and 22 (7.1%) were Fin nails. Two hundred and fifty-four (81.7%) were males and 57 (18.3%) were females giving M:F ratio of 2.7:1. The mean age was 33.68 years with minimum of 10 years and maximum of 80 years (Table 1).

**Table 1:** Summary of type of nail and sex proportion

	Nail type		Total
	SIGN nail	Fin nail	
Sex			
Male	238	16	254
Female	51	6	57
Total	289	22	311

Road traffic accident was the leading cause of long bone fractures which accounted 167 (53.7%) of 311. There were 29 (9.3%) fall down accidents and 18 (5.8%) gunshot injuries. Mechanism was unknown for 60 (19.3%) of patients (Table 2).

**Table 2:** Causes of long bone fractures and location of fracture

	Location of fracture				Total
	Proximal	Middle	Distal	Segmental	
Cause of fracture					
RTA	44	52	52	19	167
Gunshot	4	4	10	0	18
Fall down	12	9	7	1	29
Blast	0	1	0	0	1
Other	10	11	12	3	36
Unknown	19	14	26	1	60
Total	89	91	107	24	311

*RTA* road traffic accident

Two hundred and nine (67.2%) patients had femur fracture, while 100 (32.2%) had tibia fracture. There were 2 (0.6%) humeral fracture who were treated with one SIGN and the other with Fin nail. One hundred and fifty-four (49.5%) fractures were on the right side, and the rest 157 (50.5%) were on the left side. Majority of patients had simple fracture pattern 232 (42.2%) and 113 (36.3%) comminuted fractures. There were 25 (8%) segmental fracture and 41 (13.2%) wedge fractures (Table 3).

**Table 3:** Proportion of fractured bone and fracture pattern

	Fracture pattern				Total
	Simple	Wedge	Comminuted	Segmental	
Fractured bone					
Femur	94	25	77	13	209
Tibia	36	16	36	12	100
Humerus	2	0	0	0	2
Total	132	41	113	25	311

### Nature of the long bone fractures

Based on nature of fracture, 239 (76.8%) were closed and 72 (23.2%) were open. From open fractures, 26 (36.1%) were type I and 27 (37.5%) were type II. Seventeen (23.6%) were having type IIIA and 2 (2.8%) were type IIIB. There were no type IIIc fractures with SIGN nail in the study period. From 72 open fractures, time from injury to presentation and to surgery was documented in 64 patients. They presented to the hospital as early as 2 h and as late as 72 h, with mean presentation time of 13 h. Definitive SIGN nail was done at average of 13 days for open fractures**.**

### Complications after SIGN nail

All patients took prophylaxis intravenous ceftriaxone prior to surgery. All had reamed SIGN nail. Two hundred and ninety-one (93.6%) had open reduction and fixation, while 20 (6.4%) were treated with closed. Two hundred and thirty-five (75.6%) patients were approached antegrade and 76 (24.4%) were retrograde nails.

**Fig. 1:**
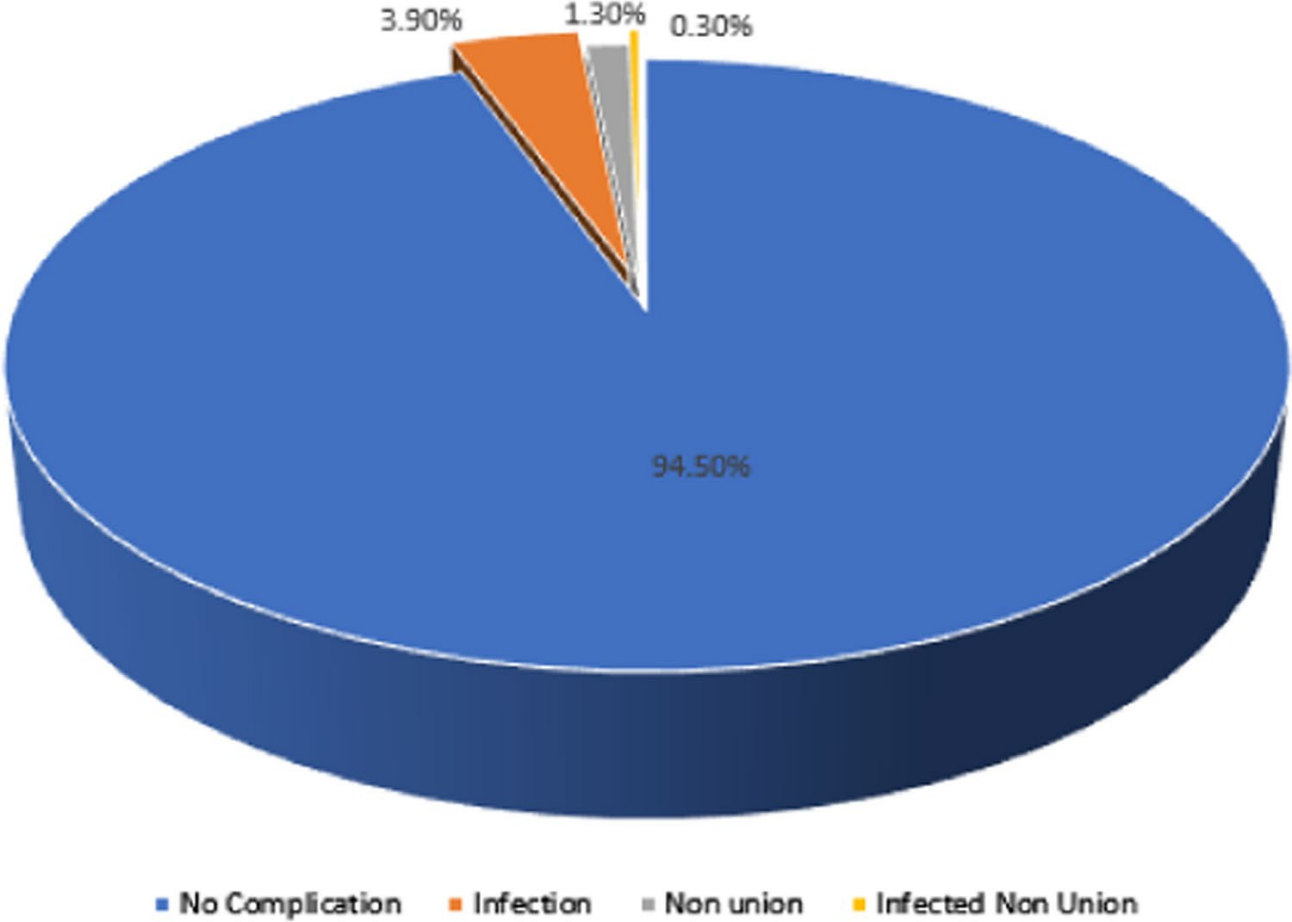
Pie chart shows complications of SIGN nail

The mean follow-up was 21.5 weeks with a minimum follow-up of 2.5 weeks and maximum follow-up of 142.5 weeks. Two hundred and ninety-four (94.5%) of patients had no complications, while 17 (5.5%) had complications. Thirteen (4.2%) patients had infection, and one was with septic nonunion. Ten (3.2%) of all long bone fractures were diagnosed to have chronic osteomyelitis, while 2 (0.6%) were deep infections and one (0.3%) had superficial surgical site infection only (Fig. 1).

Thirteen (13) patients had developed infection after SIGN nailing (Table 4).

**Table 4:** Case summery of infected SIGN nails

S.no	Age	Sex	Cause of fracture	Bone type	Pattern	Nature	Nail type	Reduction technique	Infection type
1	60	F	Other	Femur	Simple	Closed	SIGN	Open	Osteomyelitis
2	28	M	RTA	Tibia	Comminuted	Closed	SIGN	Open	Deep
3	26	M	Gunshot	Femur	Simple	IIIA	SIGN	Open	Deep
4	27	M	RTA	Femur	Segmental	Closed	SIGN	Open	Osteomyelitis
5	60	F	RTA	Tibia	Simple	Closed	SIGN	Open	Osteomyelitis
6	23	F	RTA	Femur	Comminuted	Closed	SIGN	Open	Osteomyelitis
7	50	F	RTA	Tibia	Simple	Closed	SIGN	Closed	Osteomyelitis
8	25	M	Other	Tibia	Simple	Closed	SIGN	Open	Osteomyelitis
9	28	M	RTA	Tibia	Comminuted	Type II	SIGN	Open	Osteomyelitis
10	22	M	RTA	Femur	Simple	Closed	SIGN	Open	Osteomyelitis
11	22	F	Other	Femur	Comminuted	Closed	SIGN	Open	Osteomyelitis
12	13	M	Fall	Femur	Comminuted	Closed	SIGN	Open	Osteomyelitis
13	35	M	Unknown	Tibia	Simple	Closed	SIGN	Open	Superficial

*F* female, *M* male, *RTA* road traffic accident

As it was shown on above Table 4, there were 13 cases out of 311 SIGN cases who had infection. The mean age from infected group was 30.5 years with minimum of 13 and maximum of 60. There were five females and seven males in the infected SIGN group. Based on the mechanism, there were seven RTA, three others, one fall, one gunshot and one was unknown. Seven infections were femur, and six were tibial infections, and there was no infected humerus. Considering fracture pattern seven were simple, five were comminuted and one was segmental fracture. Eleven infections were closed, and the rest two were open fractures. One of the open fractures who developed infected SIGN was a 26-year-old male who sustained gunshot injury (GA type IIIA). The other was a 28-year-old male after RTA (GA type II). All were SIGN nails, and there was no infected Fin nail. Open reduction was done for all except one for whom closed reduction was done. Ten patients developed osteomyelitis, two deep infections and one superficial infection.

## Discussion

Long bone fractures are severe injuries commonly resulting from high-energy trauma, usually due to road traffic collisions. Long bone post-traumatic osteomyelitis (PTOM) is a relatively frequent complication following surgical fixation of long bone fractures that poses many complex challenges [31]. This study aimed to assess the prevalence infection among patients who had long bone fractures and treated with SIGN nailing. We had analyzed 311 cases by taking age, sex, nature of fracture, pattern of fracture, mechanism of injury, follow-up in weeks, nail type, reduction technique and presence or absence of infection as predictors of clinical outcome and in our study we found that the prevalence infection among patients who had long bone fractures and treated with SIGN nailing was 4.2%. This finding is higher compared with a study conducted in UK which showed that the overall fractured related infection rate was 1.16% [32]. This might be due to setup difference; developing versus developed countries. On the other hand, our study had lower prevalence of surgical site infection (SSI) compared with another study conducted in Brazil which noted that the prevalence of surgical site infection (SSI) after intramedullary nailing of femoral and tibial diaphyseal fractures was 11.8% [5]. The discrepancy might be due to the previous study included potential risk factors for infection like immunosuppressive conditions. The other study also showed that the overall infection rate was 10.1% after intramedullary nailing for both femur and tibial fractures [33]. Our study result lower than this study. Could be reasoned out our study analyzed small sample size compared with the previous study.

The mean age and the gender distribution of the patients confirm that trauma remains a problem of the active young males. In this study, the mean age was 33.68 with male to female ratio of 2.7:1. This study coincides with other studies, in Nigeria male to female ratio of 2:1 with mean age of 28.6 [34]. This was also true with another study in Ethiopia, which was done in Black Lion hospital showed man age of 33 years and M:F of 3:1 [6].

Road traffic accident was the leading cause of injury which was 167 (53.7%). In Nigeria it accounted 75.3%, 657 out of 873 patients [34]. A study in Ethiopia showed leading cause of the injuries was road traffic crash 125 of 166 (75.3%) [6]. Fracture-related infection (FRI) is one of the most challenging complications following operative management of fractures [35].

Post-traumatic infection rate of long bone fractures varies depending on the severity of the injury. Prevalence of infection following internal fixation of fractures is approximately 5% overall but may exceed 30% in open fractures [29]. In contrast, the infection rate following intramedullary nailing of closed fractures is approximately 1–3% [6].

In one study in Ethiopia, infection rate of open fracture was 13.6% and 1.3% for closed fractures [6]. Our overall infection rate (4.2%) is almost similar with the surgeries done in the developed countries (5%) [29], but infection rate for open fracture was 2.78% which is lower than the other studies. This is probably due to lack of reporting, and we might be more conservative to treat open fractures with SIGN nail. Prevalence of infection for closed fracture was 4.6%, which is a bit higher.

Regarding the type of infections, there were a total of 13 (4.2%) infections out of 311 cases. Of which, 10(3.2%) had osteomyelitis, 2 (0.6) developed deep infection and 1 (0.3%) had only superficial infection. Another study in Nigeria, 42 of the fracture (4.8%) developed infection including superficial wound infections 16 (1.8%), deep wound infections, 12 (1.4%) and chronic osteomyelitis 14 (1.6%) out of 873 cases. Superficial and deep infections were a bit lower than osteomyelitis. This might be justified by under reporting of these infections.

## Conclusions

SIGN nail showed excellent results, with minimal complication rates which significantly improves our fracture care by reducing the complication, shortened hospital stays, early patient mobility, and patient return to normal function early.

We conclude that the overall prevalence of infection in our SIGN intramedullary nailing is almost similar surgeries done in the developed countries. Because of the limited data and few numbers of cases, it was difficult to do associations of the outcome variable with the predictor variable.

### Limitations

The limitations of this study were: small number of cases, being retrospective cross-sectional study and incomplete SIGN surgical data sources. Because of the limited data and few numbers of cases, it was difficult to do associations of the outcome variable with the predictor variables.

### Recommendations

We recommend the future researchers to focus their title comparing infection rate between SIGN nail and Küntscher nail. As long as there is no significant contamination and proper debridement is done, we recommend to fix more open long bone fractures with IM nailing than being conservative. To see the associations, we also recommend a large sample size with prospective study design.
